# Energy-Efficient Transmissions for Remote Wireless Sensor Networks: An Integrated HAP/Satellite Architecture for Emergency Scenarios

**DOI:** 10.3390/s150922266

**Published:** 2015-09-03

**Authors:** Feihong Dong, Hongjun Li, Xiangwu Gong, Quan Liu, Jingchao Wang

**Affiliations:** 1 College of Communications Engineering, PLA University of Science and Technology, 88 Houbiaoying Rd., Nanjing 210007, China; E-Mails: xclhj1985@163.com (H.L.); yiheng689@126.com (X.G.); 2 Institute of China Electronic System Engineering Corporation, 13 Dacheng Rd., Beijing 100141, China; E-Mails: liuquan61s@163.com (Q.L.); wangjc_61@163.com (J.W.)

**Keywords:** remote wireless sensor networks, emergency communications, integrated high altitude platform/satellite architecture, path selection, energy-efficient transmissions, link-state advertisement

## Abstract

A typical application scenario of remote wireless sensor networks (WSNs) is identified as an emergency scenario. One of the greatest design challenges for communications in emergency scenarios is energy-efficient transmission, due to scarce electrical energy in large-scale natural and man-made disasters. Integrated high altitude platform (HAP)/satellite networks are expected to optimally meet emergency communication requirements. In this paper, a novel integrated HAP/satellite (IHS) architecture is proposed, and three segments of the architecture are investigated in detail. The concept of link-state advertisement (LSA) is designed in a slow flat Rician fading channel. The LSA is received and processed by the terminal to estimate the link state information, which can significantly reduce the energy consumption at the terminal end. Furthermore, the transmission power requirements of the HAPs and terminals are derived using the gradient descent and differential equation methods. The energy consumption is modeled at both the source and system level. An innovative and adaptive algorithm is given for the energy-efficient path selection. The simulation results validate the effectiveness of the proposed adaptive algorithm. It is shown that the proposed adaptive algorithm can significantly improve energy efficiency when combined with the LSA and the energy consumption estimation.

## 1. Introduction

Emergency scenarios can benefit from the deployment of a remote wireless sensor network (WSN) in the target area for a two-fold task: (1) gathering important information from the field; and (2) supporting audio, video and data communication when other terrestrial systems are not available. Additionally, sensor devices are frequently complemented by additional multimedia traffic sources (*i.e.*, laptop computers, cameras and smart phones) [1].

Emergency communications can provide information transfer services for rescuers and victims in disasters using various sensor devices through a remote WSN [2]. The capability to exchange information (e.g., video, voice and data) is essential to improve the coordination of rescuers during an emergency crisis and the response efforts.

Due to extreme conditions, high altitude platforms (HAPs) [3] and satellites [4] are vastly underused and can be utilized for various uses to construct a remote WSN. Integrated HAP/satellite (IHS) networks are expected to optimally meet the emergency communication requirements of emergency relief and recovery operations for tackling large-scale natural and man-made disasters [5]. IHS networks have a number of potential advantages over conventional technologies [6], including:

- High capacity regional coverage;
- Rapid deployment;
- Low power consumption;
- Potentially low cost.

The IHS network infrastructure can indeed provide services for a wide range of distributed sensor devices with adapted and scalable access network capacity and coverage, owing to the low delay and high capacity HAP links [7], while reliable backhauling links to remote networks are supplied by the satellite segment [8].

## 2. Motivation and Related Works

Various platforms can be used for the HAPs depending on the telecommunication mission timescales. A stratosphere airship will be the likely choice for missions with timescales of months or years, while for missions over several days or weeks, an unmanned aerial vehicle (UAV) may be more suitable or a manned plane for a mission with a timescale of hours. For example, the high altitude airship (HAA) of Lockheed Martin, which is a kind of stratosphere airship, has a planned mission time of one month [9]. Solar energy is considerably appealing to provide communication services over a timescale of months or years, particularly if it is assumed that, for either buoyancy or aerodynamic lift in the thin atmosphere, the HAP will contain large surfaces suitable for collection [10]. At the Equator, the solar power flux can reach up to 1300 $W/m^{2}$, which is quite adequate for an HAP energy source, even if solar cell efficiencies of 10%–15% are assumed. However, the problem remains that energy has to be stored for overnight use. At higher latitudes or during winter months, the available power for overnight use will not be sufficient. Adding batteries, such as lithium-ion at about 110 W·h/kg, will result in a very large, weighty and expensive HAP [11]. Battery power is generally considered for emergency communications for small-sized UAVs with timescales of days or weeks. Therefore, the HAP is not only a power-limited system, but it is also in some cases an energy-limited system, in contrast with the satellite platform. Since the HAPs are battery-powered units, energy-efficient transmission over the IHS network is important, which also includes the battery-operated remote sensor devices. Since there is a clear requirement to operate under energy-constrained situations, the power should be used more efficiently through careful network optimization and energy-efficient transmission schemes.

In recent years, emergency communication networks and energy-efficient transmissions have undergone extensive studies within WSNs. We briefly sum the related works up into three topics: the emergency network architectures, energy-efficient transmissions and integrated HAP/satellite networks, which are discussed further in the following subsections.

### 2.1. Emergency Network Architectures

Emergency network architecture deployment has been previously studied for the provision of wireless communications and related services. Casoni *et al.* [12] have proposed an emergency network architecture, which is based on the integration of satellite and Long-term Evolution (LTE) networks for both infrastructure-based and infrastructure-less scenarios. The LTE services were brought to the disaster area using deployable mobile units and through a satellite backhaul. The architecture can provide easy connectivity in both indoor and outdoor scenarios in disasters, extended coverage and high performance guarantees, without requiring extensive configuration. Deaton [13] considered that natural disasters and terrorist acts had significant potential to disrupt ground emergency communication systems, including first-responder, cellular, landline and emergency answering services, such as 911. He concluded that the HAP network architecture could be fitted with telecommunications equipment and used to support these critical communications missions when a catastrophic event occurred. Asensio *et al.* [14] presented a smart emergency signaling system architecture for tunnels and large infrastructures in smart city scenarios, which significantly improves the actual functionality of the signal. Aranti *et al.* [15] investigated how the HAP network architecture effectively supports multimedia broadcast/multicast service (MBMS) in a scenario where the existing terrestrial network infrastructure is not available. A radio resource management (RRM) technique was proposed for implementation in a multi-hop scenario where mobile *ad hoc* networks (MANETs) cooperated with an HAP system towards a common goal of enhancing the access to MBMS services. Lin *et al.* [16] described the design and implementation of a proof-of-concept prototype, named the active disaster response system (ADRS), which automatically performs emergency tasks when an earthquake happens. In [12,13,14,15,16], the authors provide a number of different architectures for emergency networks. However, there are quite a few works in the literature that emphasize the IHS architecture’s suitability for emergency communications.

### 2.2. Energy-Efficient Transmissions

Elhawary *et al.* [17] proposed a cooperative communication protocol for energy-efficient transmission, which considers a cluster of sensor devices cooperating together in WSNs. Nasim *et al.* [18] presented an energy-efficient transmission strategy for WSN deployments over large geographical areas. The performance is enhanced by cooperative multiple-input multiple-output (MIMO). However, [17,18] did not consider the role of the HAP. By considering a single HAP architecture, Kandeepan *et al.* [19] discussed the design and evaluation of an adaptive cooperative scheme intended to extend the survivability of the battery-operated aerial-terrestrial communication links. He emphasized that the cooperation between mobile terrestrial terminals on the ground could improve energy efficiency in the uplink. By considering a satellite architecture, Alagoz *et al.* [20] presented and discussed networking from the perspective of energy efficiency. Satellite communications can become drastically more energy efficient if the constituent systems can exploit their inherent energy-efficiency-related advantages. However, [19,20] did not consider more practical multiple HAP/satellite scenarios. Zhu *et al.* [21] and Madan *et al.* [22] developed optimal and approximately optimal energy-efficient transmission strategies with the objective of either minimizing the outage probability or maximizing the ergodic rate in a Rayleigh fading environment. However, in our work, the channels of the HAPs to the terminals and the channels of the satellite to the terminals are Rician fading rather than Rayleigh fading [23,24].

### 2.3. Integrated HAP/Satellite Networks

As mentioned at the end of Section 2.1, there are relatively few studies on the IHS architecture for energy-efficient transmissions in emergency communications. However, there are articles about the IHS networks for other related aspects in WSNs. In [4], the authors presented a review of the IHS networks in WSNs and demonstrated the technological advantages of the IHS networks for a wide range of monitoring, Earth observations and other sensing-based applications. However, the authors did not highlight the energy-efficiency-related problems. The energy constraint is crucial for battery-powered HAPs and remote sensor devices. In [25], the authors proposed schemes for an IHS-based network for delivering emergency calls and multicast and broadcast services (MBS) in case of large-scale disasters in mountainous areas. Appropriate standard options were chosen, and necessary adaptations were recommended by considering the special requirement of delivering emergency communication services from the HAP. Wang *et al.* [26] presented a heterogeneous network system comprised of a mission layer, an HAP layer and a satellite layer. The energy consumption was minimized using a deterministic annealing algorithm. Luglio *et al.* [27] presented an innovative call admission control (CAC) scheme that uses transmission control protocol (TCP) statistics as one of its inputs and is able to manage different classes of users for an IHS communication system in emergency situations. Pace *et al.* [28] show how space technologies and new integrated telecommunication networks mitigate the impact of natural and man-made disasters. Potentially attractive telecommunication architectures to better manage a disaster scenario were discussed. Therefore, the existing studies have shown that IHS can offer a promising communication architecture for emergency scenarios.

Although the deployments and demonstrations of emergency communications have been extensively investigated, many issues and research aspects regarding the possible integration of different architectures and technologies still have open questions that require deeper investigation. Quite a few works in the literature have addressed the issue of energy-efficient transmissions in the IHS architecture for emergency scenarios. As it is crucially important for the theoretical research and application of emergency communications, this paper will address the energy-efficient strategies and algorithms in an IHS architecture for an emergency scenario. The simulation will further prove that the proposed IHS architecture and transmission strategy are promising solutions for emergency communications.

### 2.4. Contributions

The main contributions that this work addresses, which have not been included in previous studies, are as follows:

- An IHS architecture for emergency scenarios is proposed for the first time, which consists of three segments. The architecture is explained in detail, and we conclude that the space segment, near space segment and ground segment will work closely together to provide information transfer services for remote sensor devices.
- The transmission power requirements of the terminal end and HAP end are investigated in a slow flat Rician fading channel. The gradient descent and differential equation methods are used to obtain the optimal transmitted power while maintaining a minimum bit error rate (BER) level.
- Using the concept of link-state advertisement (LSA), a novel energy-efficient transmission strategy for the energy-efficient path selection is designed. The strategy can significantly reduce the energy consumption, both of the terminal and HAP, which is demonstrated through in-depth numerical simulations.

The remainder of this paper is organized as follows. Section 3 gives the system model, including three segments and the channel modeling. Section 4 gives the mathematical formulation of the optimization problem. The transmission power requirements are derived by the gradient descent and differential equation methods. In Section 5, we design the energy-efficient transmission strategies, which use the LSA concept. The energy consumption at the source and system level are modeled, and an adaptive algorithm for the energy-efficient path selection is given. Section 6 shows numerical simulations and results. Finally, Section 7 concludes the paper with a summary and a discussion of future research directions. Table 1 lists some key notations used in the paper.

**Table 1:** Notations.

Notation	Description	Notation	Description
$C_{i}$	The channel *i*	$n_{i}$	The path loss exponents of the channel *i*
$G_{r}$	The receive antenna gain	$k_{i}$	The Rician factor of send-receive (S-R) channel
$G_{s}$	The transmit antenna gain	$d_{i}$	The send-receive (S-R) distances
$R_{b}$	The data rate	α	The mean channel power gain
$\rho_{e}$	The bit error rate	$E_{b}$	The energy of per bit
ψ	The longitude	ϕ	The latitude
$P_{s}$	The transmission power	$\Delta \left(\cdot \right)$	The gradient operator
*E*	The energy consumption	$v_{i}^{{}_{opt.}}$	The optimal transmission path of the *i*-th terminal

## 3. System Model

**Figure 1:**
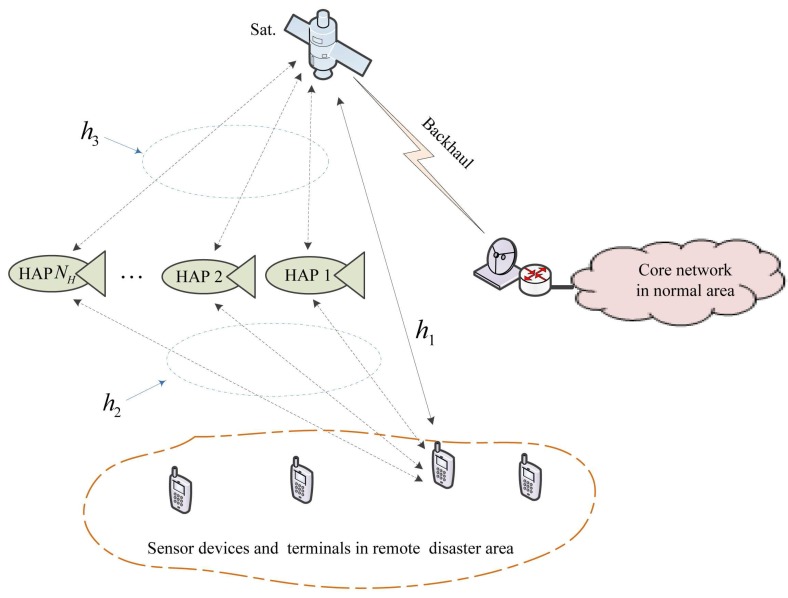
System model of the integrated high altitude platform (HAP)/satellite (IHS) network for emergency scenarios.

The system model of the IHS network for emergency communications is depicted in this section. When a disaster occurs, an emergency communication system will be established, as shown in Figure 1, which consists of three parts, *i.e.*, space segment, near space segment and ground segment. The details of the three segments are described in the following subsections.

### 3.1. Space Segment

The space segment consists of a satellite in geosynchronous (GEO) orbit. This satellite is a general purpose satellite in another communication system, e.g., a commercial service and civil service satellite system. It is temporarily rented for emergency communications rather than being a specified satellite, which is researched, developed and launched for emergency communications [29]. Therefore, the emergency system will be low cost. The functions of the space segment mainly include four aspects:

- Data back to the core network, *i.e.*, the Ka/Ku band or laser backhaul link [30];
- Emergency links for the rescuers’ and victims’ terminals, *i.e.*, the L/Sband links [20];
- Relay links from the HAPs, *i.e.*, the Ka/Ku band or laser relay links [8];
- Broadcasting the LSA for the HAPs and terminals. The detail of the LSA is given in Section 4.1.

### 3.2. Near Space Segment

The near space segment consists of a number of HAPs. In general, the HAPs are urgently launched across the disaster area. The number of launched HAPs usually depends on the communication requirements. Therefore, the main cost of the emergency communication system is the cost of the HAPs in our proposed system model. Due to its inexpensive reusable platform (*circa* millions of dollars) compared to a costly satellite [31], a large coverage area and easily rapid deployment compared to the ground base station, the HAP is cost-effective. The payloads of the HAP contain two parts:

- LTE/WiMAXcommunication payload for the user links [32];
- Communication payload supporting the Ka/Ku band or laser relay links to the satellite [8].

The most promising energy subsystems for battery-powered HAPs are the regenerative fuel cell (RFC) and the electrical battery. In fact, most HAP programs worldwide are considering the use of the RFC onboard HAPs for unlimited operation endurance. Battery power is particularly suited to small-sized UAVs with timescales of days or weeks. Furthermore, as mentioned in Section 2, for HAPs with a solar panel, any surplus of energy captured by solar cells during the day can be stored in electrical batteries or in RFCs.

### 3.3. Ground Segment

The ground segment consists of two sub-segments:

- User sub-segment: the sensor devices and terminals of rescuers and victims in the remote disaster area;
- Core network sub-segment: the service provider and national rescue institute in the normal area.

The remote sensor devices and terminals of rescuers and victims are multi-radio portable devices [33], which support the LTE/WiMAX communicating with the HAPs and the DVB-RCS communicating with the satellite. The core network sub-segment is responsible for routing and exchanging information with the national rescue institute.

As a consequence, an IHS network is then available for emergency scenarios. The devices and terminals can select the relay HAPs or the GEO satellite, which is in view, to achieve an urgent information transfer. At the same time, the service provider and national rescue institute in the normal area can transmit rescue information, including voice, video and data, via the GEO satellite to the devices and terminals. In the paper, we will mainly investigate the former information transfer due to the fact that devices and terminals use more energy when transmitting a signal and less constant energy when receiving a signal.

### 3.4. Channel Model

In the IHS network scenarios, there are three types of channels. These are terminal-to-satellite (T-S) direct channels, terminal-to-HAP (T-H) relay channels and HAP-to-satellite (H-S) relay channels, which are denoted as $h_{1}$, $h_{2}$ and $h_{3}$ in Figure 1. Channel $h_{1}$ is Rician small-scale fading with serious free-space loss, a long-delay and high elevation in mid- and low-latitude regions, due to the remote communication distance between the terminal and satellite. Channel $h_{2}$ is Rician small-scale fading with mild free-space loss, a low-delay and various elevation angles, due to the relatively close distance between the terminal and the HAP. Compared to $h_{1}$, obstruction by buildings and other tall objects is serious for $h_{2}$, due to its various elevation angles. Finally, channel $h_{3}$ is almost an ideal channel with additive white Gaussian noise (AWGN), due to the quasi-vacuum between the HAP and satellite. We assume no multipath signals, resulting in a slow flat fading channel, where the fading of the channel can be considered to be constant over the packet duration, but changing over time. Since the user is motionless or moving at a walking speed in the disaster area, this is practical in our proposed model. The corresponding gains of all three channels vary over time based on the spatial separation between the nodes. Then, the three channels can be uniformly represented as:

(1) $$C_{i}=f\left(n_{i},d_{i},\kappa_{i},h_{i}\right),i=1,2,3$$

where $n_{i}$ is the path-loss exponent, $d_{i}$ denotes the send-receive (S-R) distance, $\kappa_{i}$ denotes the Rician factor and $h_{i}$ is the small-scale slow flat fading gain. For channel $h_{3}$, the Rician factor is ideally $\kappa_{3}=\infty$. The values of $n_{i}$ depend on the specified propagation environment. For example, in free space, the value of $n_{i}$ is equal to two, and when obstructions are present, $n_{i}$ will have a lager value. By using $d_{i}$, the average path loss can be calculated as $L_{m}\left(d_{i}\right)=L_{f}\left(d_{0}\right){\left(d_{i}/d_{0}\right)}^{n_{i}}$, where $d_{0}\leq d_{i}$ is the reference distance. $L_{f}\left(d_{0}\right)={\left(4\pi fd_{0}/c\right)}^{2}$ is the free-space path-loss with reference distance $d_{0}$, where *f* is the carrier frequency and $c=3\times 10^{8}\;m/s$ is the speed of light. Therefore, the overall characteristics of channel *i* are then a function of $n_{i},d_{i},\kappa_{i}$ and $h_{i}$.

Assuming the transmitted signal is s(t), the received signal $r_{i}\left(t\right)$ can be given by:

(2) $$r_{i}\left(t\right)=\frac{1}{\sqrt{L_{m}\left(d_{i}\right)}}h_{i}\left(t\right)s\left(t\right)+n\left(t\right)$$

where n(t) denotes the corresponding AWGN with a double-sided power density of $N_{0}/2$ at the receiving end. As shown in Equation (2), we consider that there are the same power spectral densities for all of the receivers in the three segments above for simplicity.

The mean signal-to-noise ratio (SNR) of the received signal is given by $\gamma =\alpha E_{b}/N_{0}$, where $E_{b}\;=\;P_{s}G_{s}G_{r}/\left(R_{b}L_{m}\left(d_{i}\right)BO_{o}\right)$ denotes the energy per bit. $G_{s}$, $G_{r}$ and $\alpha =\frac{1}{t}\int_{0}^{t}h^{2}\left(t\right)dt$ are the transmitter antenna gain, receiver antenna gain and mean fading channel power gain, respectively. $R_{b}$ (bit/s) denotes the data rate. $BO_{o}$ denotes the back-off of the output power. If differential phase-shift keying (DPSK) modulation is used, the corresponding bit error rate (BER) for the T-S direct channel and T-H relay channel is given in [34,35] by:

(3) $$\rho_{e}=\int_{0}^{\infty }P_{e}\left(X\right)p\left(X\right)dX=\frac{\left(1+\kappa \right)}{2(1+\kappa +\gamma )}\exp\left(\frac{-\kappa \gamma }{1+\kappa +\gamma }\right)$$

where $X=\beta^{2}E_{b}/N_{0}$ denotes the temporal SNR at the receiving end, $P_{e}\left(X\right)=\frac{1}{2}\exp\left(-X\right)$ is the BER for DPSK in the AWGN channel, $p\left(X\right)=\frac{1+\kappa }{\gamma }\exp\left(-\frac{X(1+\kappa )+\kappa \gamma }{\gamma }\right)K_{0}\left(\sqrt{\frac{4(1+\kappa )\kappa X}{\gamma }}\right)$ is the Rician probability density function (PDF) in terms of *X*, $K_{0}\left(\cdot \right)$ denotes the zero order modified Bessel function of the first kind and β denotes the temporal gain of the fading channel.

## 4. Mathematical Formulation of the Optimization Problem

Unlike traditional strategies, which always sense wireless radio environments at the terminal end, the energy-efficient transmission strategy proposed in this paper releases the link quality information by broadcasting the LSA in the HAP and satellite ends without measurement at the terminal end. This will potentially reduce the energy cost of the signal processing and improve the energy efficiency at the terminal end, which is very effective for the slow flat fading channels. The mathematical formulation of the optimization problem for the proposed model is given in the following sections.

### 4.1. Link-State Advertisement

LSA is produced in the space segment and the near space segment, *i.e.*, the HAPs and the satellite. During the periodic LSA broadcast, each of the HAPs and the satellite broadcasts its address, geographic position (longitude $\psi_{j}$ and latitude $\phi_{j}$), transmission power $P_{s,j}$ and antenna gain $G_{s,j}$ to the users. Although the terminal has no requirement to sense the wireless radio environment, the reception and processing of the LSA still uses additional energy. Therefore, the size of the advertisement packets should be kept to a bare minimum to ensure no unnecessary energy is consumed during the advertisement receiving periods.

When the LSA of the *j*-th HAP or satellite is received by the *i*-th terminal, by combining this with its own location (longitude $\psi_{i}$ and latitude $\phi_{i}$), the distance between the *i*-th terminal and *j*-th node is given in [36] by:

(4) $$d_{i,j}=\sqrt{{\left(l_{i}+R\right)}^{2}+{\left(l_{j}+R\right)}^{2}-2\left(l_{i}+R\right)\left(l_{j}+R\right)\cos\theta_{i}}$$

where *R* denotes the Earth’s radius, $l_{i}$ and $l_{j}$ denote the altitude of *i*-th terminal and *j*-th nodes and $\theta_{i}=\arccos\left(\cos\left(90-\phi_{i}\right)\cos\left(90-\phi_{j}\right)+\sin\left(90-\phi_{i}\right)sin\left(90-\phi_{j}\right)\cos\left(\psi_{i}-\psi_{j}\right)\right)$ are the azimuth angles.

When $d_{i,j}$ is obtained, for an S-R link, $L_{m}\left(d_{i,j}\right)$ will be calculated, and the temporal gain β of fading can be estimated by computing the received power level of the reporting packet and using the knowledge about the LSAs (e.g., transmission power $P_{s,j}$ and antenna gain $G_{s,j}$).

### 4.2. Transmission Power Requirements for the T-S Link

In this subsection, we try to solve the transmission power requirements of the terminal for the T-S direct link. We consider the BER expression in Equation (3). Obviously, an explicit expression of the SNR cannot be obtained due to the fact that Equation (3) is a composite function of a polynomial and exponential. However, the BER $\rho_{1}$ of the T-S link is a strictly decreasing function in the domain of the transmission power $P_{s,1}$. Therefore, for the BER to maintain a minimum level ϵ of service quality, $P_{s,1}$ of the terminal can be iteratively computed using the gradient descent method for some $\xi_{1}>0$ and $\rho_{e}=\epsilon$, shown as:

(5) $${\hat{P}}_{s,1,n+1}={\hat{P}}_{s,1,n}-\xi_{1}\nabla \left({\hat{P}}_{s,1,n}\right)$$

where $\nabla ({\hat{P}}_{s,1,n})$ is the gradient of the transmission power $P_{s,1}$, ${\hat{P}}_{s,1}$ is the desired minimum transmission power of the terminal for the T-S direct link and *n* is the iteration number.

The gradient $\nabla ({\hat{P}}_{s,1,n})$ can be derived as follows:

If the transmission power of the terminal is $P_{s,1}$, the mean received SNR for the satellite can be represented as:

(6) $$\gamma_{1}=\frac{\alpha_{1}E_{b}}{N_{0}}=\frac{\alpha_{1}P_{s,1}G_{s,1}G_{r,1}}{R_{b,1}L_{m}\left(d_{1}\right)N_{0}BO_{o}}$$

Substituting Equation (6) into Equation (3), the BER for the T-S direct transmission is given by:

(7) $$\begin{array}{c} \rho_{e,1}=\frac{\left(1+\kappa_{1}\right)}{2(1+\kappa_{1}+\gamma_{1})}\exp\left(\frac{-\kappa_{1}\gamma_{1}}{1+\kappa_{1}+\gamma_{1}}\right)\\ \;\;\;\;\;\;=B\exp\left(\frac{-\kappa_{1}\alpha_{1}P_{s,1}G_{s,1}G_{r,1}}{R_{b,1}L_{m}\left(d_{1}\right)N_{0}BO_{o}\left(1+\kappa_{1}+\frac{\alpha_{1}P_{s,1}G_{s,1}G_{r,1}}{R_{b,1}L_{m}\left(d_{1}\right)N_{0}BO_{o}}\right)}\right) \end{array}$$

where $B=\frac{1+\kappa_{1}}{2(1+\kappa_{1}+\gamma_{1})}$.

Then, the gradient of $P_{1,n}$ can be obtained as:

(8) $$\begin{array}{c} \nabla \left({\hat{P}}_{s,1,n}\right)=\frac{d(\epsilon -\rho_{e}\left(P_{s,1}\right))}{dP_{s,1}}\\ \;\;\;\;\;\;\;\;\;\;\;\;=-\frac{dB}{dP_{s,1}}\exp\left(\frac{-\kappa_{1}\gamma_{1}}{1+\kappa_{1}+\gamma_{1}}\right)-B\frac{d\exp\left(\frac{-\kappa_{1}\gamma_{1}}{1+\kappa_{1}+\gamma_{1}}\right)}{dP_{s,1}}\\ \;\;\;\;\;\;\;\;\;\;\;\;=\frac{\left(1+\kappa_{1}\right)}{2{\left(1+\kappa_{1}+\gamma_{1}\right)}^{2}}\frac{\alpha_{1}G_{s,1}G_{r,1}}{R_{b,1}L_{m}\left(d_{1}\right)N_{0}BO_{o}}\exp\left(\frac{-\kappa_{1}\gamma_{1}}{1+\kappa_{1}+\gamma_{1}}\right)+B\exp\left(\frac{-\kappa_{1}\gamma_{1}}{1+\kappa_{1}+\gamma_{1}}\right)\times \frac{\kappa_{1}\left(1+\kappa_{1}\right)}{{\left(1+\kappa_{1}+\gamma_{1}\right)}^{2}}\times \frac{\alpha_{1}G_{s,1}G_{r,1}}{R_{b,1}L_{m}\left(d_{1}\right)N_{0}BO_{o}}\\ \;\;\;\;\;\;\;\;\;\;\;\;=\frac{\alpha_{1}G_{s,1}G_{r,1}}{R_{b,1}L_{m}\left(d_{1}\right)N_{0}BO_{o}}\left(\frac{B}{\left(1+\kappa_{1}+\gamma_{1}\right)}+\frac{\kappa_{1}\left(1+\kappa_{1}\right)B}{{\left(1+\kappa_{1}+\gamma_{1}\right)}^{2}}\right)\exp\left(\frac{-\kappa_{1}\gamma_{1}}{1+\kappa_{1}+\gamma_{1}}\right)\\ \;\;\;\;\;\;\;\;\;\;\;\;=\frac{\alpha_{1}G_{s,1}G_{r,1}B\left(2B\kappa_{1}+1\right)}{L_{1,m}\left(d_{1}\right)N_{0}BO_{o}R_{b,1}\left(1+\kappa_{1}+\gamma_{1}\right)}\exp\left(\frac{-\kappa_{1}\gamma_{1}}{1+\kappa_{1}+\gamma_{1}}\right) \end{array}$$

Therefore, the gradient $\nabla ({\hat{P}}_{1,n})$ can be represented as:

(9) $$\nabla \left({\hat{P}}_{s,1,n}\right)=\frac{\alpha G_{s,1}G_{r,1}B\left(2B\kappa_{1}+1\right)}{L_{1,m}\left(d_{1}\right)N_{0}BO_{o}R_{b,1}\left(1+\kappa_{1}+\gamma_{1}\right)}\times \exp\left(\frac{-\kappa_{1}\gamma_{1}}{1+\kappa_{1}+\gamma_{1}}\right)$$

Therefore, the minimum transmission power of the terminal for the T-S direct link ${\hat{P}}_{s,1}$ can be calculated by performing a sufficient number of iterations *n* in Equation (5).

### 4.3. Transmission Power Requirements for Terminal-HAP-Satellite Path

For the terminal-to-HAP and the HAP-to-satellite (T-H-S) path, *i.e.*, the indirect or relay link, if it assumed that the decode-and-forward (DF) is used at the HAP end, then the overall BER of the relay link can be given by:

(10) $$\rho_{e,r}=\left(1-\rho_{e,2}\right)\rho_{e,3}+\left(1-\rho_{e,3}\right)\rho_{e,2}$$

where $\rho_{e,2}$ denotes the BER of the T-H relay link and $\rho_{e,3}$ denotes the BER of the H-S relay link. $\rho_{e,2}$ can be obtained by Equation (3). Due to the AWGN channel of $h_{3}$, the BER of DPSK in the H-S link can be obtained as:

(11) $$\rho_{e,3}=\frac{1}{2}\exp\left(-\gamma_{3}\right)$$

Since the BER values are small (*circa* $10^{-6}$), the values of BER $\rho_{e,2}$ and $\rho_{e,3}$ will be much larger than that of $\rho_{e,2}\times \rho_{e,3}$. The overall BER can be approximated as $\rho_{e,r}=\rho_{e,2}+\rho_{e,3}$. Let $P_{s,2}$ and $P_{s,3}$ denote the transmission power of the terminal for the T-H relay link and the transmission power of the HAP for the H-S relay link, respectively. With the BER maintaining minimum levels $\rho_{e,3}=\epsilon_{3}$, $\rho_{e,2}=\epsilon_{2}$ and $\epsilon_{2}+\epsilon_{3}=\epsilon$, the minimum total transmission power can be obtained by:

(12) $$\left\{{\hat{P}}_{s,2},{\hat{P}}_{s,3}\right\}=arg\min_{P_{s,2}}\{P_{s,2}+P_{s,3}\}$$

where ${\hat{P}}_{s,2}$ and ${\hat{P}}_{s,3}$ denote the optimal transmission powers of the T-H link and H-S link, respectively.

According to Equation (11), the transmission power $P_{s,3}$ can be represented as:

(13) $$P_{s,3}=\frac{-R_{b,3}L_{m,3}\left(d_{3}\right)N_{0}BO_{o}}{\alpha_{3}G_{s,3}G_{r,3}}ln\left(2\left(\epsilon -\epsilon_{2}\right)\right)$$

where $\epsilon_{2}=\frac{\left(1+\kappa_{2}\right)}{2(1+\kappa_{2}+\gamma_{2})}\exp\left(\frac{-\kappa_{2}\gamma_{2}}{1+\kappa_{2}+\gamma_{2}}\right)$ is the BER of the corresponding T-H relay link, with $\gamma_{2}\;=\;\alpha_{2}P_{s,2}G_{s,2}G_{r,2}/\left(R_{b,2}L_{m}\left(d_{2}\right)N_{0}BO_{o}\right)$.

It can be found that $P_{s,3}$ is a function of the independent variable $P_{s,2}$. If the total transmission power is $P_{r}=P_{s,2}+P_{s,3}$, the first order derivative of $P_{r}$ can be derived as follows:

With the overall BER of the T-H-S path maintained at a minimum level $\epsilon_{2}+\epsilon_{3}=\epsilon$, the mean received SNR for the satellite can be represented as:

(14) $$\gamma_{3}=\frac{\alpha_{3}E_{b}}{N_{0}}=\frac{\alpha_{3}P_{s,3}G_{s,3}G_{r,3}}{R_{b,3}L_{m}\left(d_{3}\right)N_{0}BO_{o}}$$

Substituting Equation (14) into Equation (11), the BER for the H-S relay transmission is given by:

(15) $$\rho_{e,3}=\frac{1}{2}\exp\left(-\gamma_{3}\right)=\frac{1}{2}\exp\left(-\frac{\alpha_{3}P_{s,3}G_{s,3}G_{r,3}}{R_{b,3}L_{m}\left(d_{3}\right)N_{0}BO_{o}}\right)$$

Based on Equation (10), the BER for the H-S relay transmission is $\rho_{e,3}=\epsilon -\epsilon_{2}$, and the transmission power $P_{s,3}$ has been given by Equation (13). The BER $\epsilon_{2}$ is determined by the transmission power $P_{s,2}$ of the terminal for the T-H relay transmission, which can be written as:

(16) $$\begin{array}{c} \rho_{e,2}=\frac{\left(1+\kappa_{2}\right)}{2(1+\kappa_{2}+\gamma_{2})}\exp\left(\frac{-\kappa_{2}\gamma_{2}}{1+\kappa_{2}+\gamma_{2}}\right)\\ \;\;\;\;\;=\frac{\left(1+\kappa_{2}\right)}{2(1+\kappa_{2}+\frac{\alpha_{2}P_{s,2}G_{s,2}G_{r,2}}{R_{b,2}L_{m}\left(d_{2}\right)N_{0}BO_{o}})}\times \exp\left(\frac{-\kappa_{2}\frac{\alpha_{2}P_{s,2}G_{s,2}G_{r,2}}{R_{b,2}L_{m}\left(d_{2}\right)N_{0}BO_{o}}}{1+\kappa_{2}+\frac{\alpha_{2}P_{s,2}G_{s,2}G_{r,2}}{R_{b,2}L_{m}\left(d_{2}\right)N_{0}BO_{o}}}\right) \end{array}$$

Substituting Equation (16) into Equation (13), the first order derivative of $P_{r}=P_{s,2}+P_{s,3}$ can then be solved as:

(17) $$\begin{array}{c} \frac{dP_{r}}{dP_{s,2}}=1+\frac{dP_{s,3}}{dP_{s,2}}\\ \;\;\;\;\;\;\;=1+\frac{-R_{b,3}L_{m,3}\left(d_{3}\right)N_{0}BO_{o}}{\alpha_{3}G_{s,3}G_{r,3}}\times \frac{dln(2\left(\epsilon -\epsilon_{2}\right))}{dP_{s,2}}\\ \;\;\;\;\;\;\;=1+\frac{-R_{b,3}L_{m,3}\left(d_{3}\right)N_{0}BO_{o}}{\alpha_{3}G_{s,3}G_{r,3}}\times \frac{-2}{2(\epsilon -\epsilon_{2})}\times (\frac{-\left(1+\kappa_{2}\right)\alpha_{2}G_{s,2}G_{r,2}}{2R_{b,2}L_{m}\left(d_{2}\right)N_{0}BO_{o}{\left(1+\kappa_{2}+\gamma_{2}\right)}^{2}}\exp\left(\frac{-\kappa_{2}\gamma_{2}}{1+\kappa_{2}+\gamma_{2}}\right)\\ \;\;\;\;\;\;\;\;\;\;\;\;\;+\frac{\left(1+\kappa_{2}\right)}{2(1+\kappa_{2}+\gamma_{2})}\exp\left(\frac{-\kappa_{2}\gamma_{2}}{1+\kappa_{2}+\gamma_{2}}\right)\times \left(\frac{-\kappa_{2}\left(1+\kappa_{2}\right)}{{\left(1+\kappa_{2}+\gamma_{2}\right)}^{2}}\times \frac{\alpha_{2}G_{s,2}G_{r,2}}{R_{b,2}L_{m}\left(d_{2}\right)N_{0}BO_{o}}\right))\\ \;\;\;\;\;\;\;=1-\frac{R_{b,3}L_{m,3}\left(d_{3}\right)}{\alpha_{3}G_{s,3}G_{r,3}\left(\epsilon -\epsilon_{2}\right)}\frac{\alpha_{2}G_{s,2}G_{r,2}}{R_{b,2}L_{m}\left(d_{2}\right)}\times \frac{\left(1+\kappa_{2}\right)\left({\left(\kappa_{2}+1\right)}^{2}+\gamma_{2}\right)}{2{\left(1+\kappa_{2}+\gamma_{2}\right)}^{3}}\exp\left(\frac{-\kappa_{2}\gamma_{2}}{1+\kappa_{2}+\gamma_{2}}\right) \end{array}$$

Therefore, we get the first order derivative of the total transmission power $P_{r}=P_{s,2}+P_{s,3}$, which can be found by:

(18) $$\frac{dP_{r}}{dP_{s,2}}=1-\frac{R_{b,3}L_{m,3}\left(d_{3}\right)}{\alpha_{3}G_{s,3}G_{r,3}\left(\epsilon -\epsilon_{2}\right)}\frac{\alpha_{2}G_{s,2}G_{r,2}}{R_{b,2}L_{m}\left(d_{2}\right)}\frac{\left(1+\kappa_{2}\right)\left({\left(\kappa_{2}+1\right)}^{2}+\gamma_{2}\right)}{2{\left(1+\kappa_{2}+\gamma_{2}\right)}^{3}}\times \exp\left(\frac{-\kappa_{2}\gamma_{2}}{1+\kappa_{2}+\gamma_{2}}\right)$$

Since $\epsilon_{2}$ is a convex function in the domain $P_{s,2}$, $P_{s,3}$ will also be a convex function in the domain $P_{s,2}$. Then, the summation of $P_{r}=P_{s,2}+P_{s,3}$ becomes convex. Therefore, when Equation (18) is equal to zero, an optimal ${\hat{P}}_{s,2}$ will be calculated. By substituting ${\hat{P}}_{s,2}$ into Equations (3) and (13), a corresponding BER $\epsilon_{2}$ and the optimal ${\hat{P}}_{s,3}$ can be obtained. Therefore, the optimal overall power $P_{r}^{opt.}$ will be obtained by adding ${\hat{P}}_{s,2}$ and ${\hat{P}}_{s,3}$ together.

## 5. Energy-Efficient Transmission Strategies

### 5.1. Energy Consumption

It is assumed that broadcast packets are used and that all of the $N_{H}$ LSAs of the HAPs and the one GEO satellite are received by the terminal in the network. Over a specified time period $T\in R^{+}$, the energy consumption of the terminal for the T-S direct link can be calculated as the integral of the transmission power over that period, which can be written as:

(19) $$E_{d}=\int_{0}^{T}P_{s,1}\left(t\right)dt+e_{Rx,LSA}$$

where $e_{Rx,LSA}=P_{Rx}T_{LSA}$ represents the energy consumption to process the broadcast LSA of the satellite and $T_{LSA}$ is the total time taken for the LSA transmission in the time period *T*. $P_{Rx}$ denotes the necessary power to process the demodulation of the received radio-frequency (RF) signal, baseband signal and coding and decoding.

For the T-H-S relay path, the energy consumption consists of the T-H link transmission energy at the terminal end and the H-S link transmission energy at the HAP end, which can be respectively represented as:

(20) $$E_{T}=\int_{0}^{T}P_{s,2}\left(t\right)dt+\left(N_{H}+1\right)e_{Rx,LSA}$$

(21) $$E_{H}=\int_{0}^{T}P_{s,3}\left(t\right)dt+P_{Rx}T+e_{Tx,LSA}$$

where $e_{Tx,LSA}=P_{Tx}T_{LSA}$ is the energy consumption for broadcasting packets of the LSA and $P_{Tx}$ denotes the transmission power of the LSA from the HAP to the terminal.

Assuming that the terminal can only select one relay HAP during one time slot Δt, the overall energy consumption of the T-H-S relay path can be given by:

(22) $$\begin{array}{c} E_{r}=E_{T}+E_{H}=\int_{0}^{\Delta t}P_{s,2}\left(t\right)dt+\int_{0}^{\Delta t}P_{s,3}\left(t\right)dt\\ \;\;\;\;\;\;\;\;\;\;\;\;\;\;\;\;\;\;\;\;\;\;\;+\left(N_{H}+1\right){e_{Rx,}}_{LSA}+P_{Rx}\Delta t+e_{Tx,LSA} \end{array}$$

For the *i*-th terminal, the HAP should be selected that has the lowest energy consumption between $E_{d,i}$ of the T-S link and $E_{r,i}$ of the T-H-S path. Therefore, the optimal transmission path $v_{i}^{{}_{opt.}}$ in a single time slot can be represented as:

(23) $$v_{i}^{{}_{opt.}}=\left\{v_{i}↔E_{i}=\min\left(E_{d,i},E_{r,i}\right)\right\},i\in N_{T}$$

If the number of terminals is $N_{T}$, then the energy consumption of $N_{T}$ terminals and the energy consumption of the overall network can be respectively represented as:

(24) $$E_{T,tot.}=\sum_{j=1}^{T/\Delta t}\sum_{i=1}^{N_{T}}E_{T,i,j}$$

(25) $$E_{tot.}=\sum_{j=1}^{T/\Delta t}\sum_{i=1}^{N_{T}}E_{r,i,j}$$

### 5.2. Path Selection

In order to communicate in an energy-efficient manner with the core network, the terminal will analyze its selection to transmit via the direct path by a T-S link to the satellite or a relay path through $N_{H}$ possible relay links in the HAP segment. Algorithm 1 shows the adaptive path algorithm for energy-efficient transmissions designed for our proposed model based on the above analysis. The inputs of the algorithm are the LSAs of the $N_{H}$ HAPs and the satellite, the required BER ϵ and the required data rate $R_{b}$, while the outputs of the algorithm are the corresponding optimal transmission power $P_{r}^{opt.,i,j}\left(t\right)$, minimum energy consumption $E_{r,i}^{j^{opt.}}$ and optimal path $v_{i}^{{}_{opt.}}$. We can observe that, for each time slot, the terminal sends the signal with the optimal power $P_{r}^{opt.,i,j}\left(t\right)$ via an optimal path $v_{i}^{{}_{opt.}}$ and consumes minimum energy $E_{r,i}^{j^{opt.}}$.

**Algorithm 1.** Adaptive algorithm for energy-efficient path selection.**Inputs:** LSAs, required BER ∊, required data rate $R_{b}$**Outputs:** Optimal transmission power $P_{r}^{\mathrm{opt.},i,j}(t)$, minimum energy consumption $E_{r,i}^{j^{\mathrm{opt.}}}$, optimal path $v_{i}^{\mathrm{opt.}}$
1:**if** *data(k)* is required to send in the *i*-th terminal, *i.e.*, information source $T_{i}$2: Calculate $d_{i,j}$ based on the LSAs and Equation (4);3: Calculate $P_{s,j}^{i}(t)$ of the T-S link for a given BER ∊ using Equation (5);4: Calculate $P_{r}^{\mathrm{opt.},i,j}(t)={\hat{P}}_{s,2}^{i,j}(t)+{\hat{P}}_{s,3}^{i,j}$ of the *j*-th T-H-S path for all possible HAPs (*j* = 1, 2, ⋯ ,$N_{H}$) for a given BER ∊ using Equations (18) and (13);5: Obtain the energy consumption $E_{d,i}$ and $E_{r,i,j}$ for each path using Equations (19) and (22);6: Select the relay path with minimal energy consumption $E_{r,i}^{j^{\mathrm{opt.}}}=\min\{E_{r,i,j}|j=1,2,\cdots \;,N_{H}\}$;7:**if** $E_{d,i}$ ≤ $E_{r,i}$ **then**8: Select the T-S direct path, *i.e.*, transmission power $Tx=P_{s,1}^{i}$ to satellite;9: Wait for *data(k+1)*;10:**else**11: Select the $j^{\mathrm{opt.}}$-th T-S relay path, *i.e.*, *i*-th terminal transmission power $Tx_{T}={\hat{P}}_{s,2}^{i,j}$ to HAP, $j^{\mathrm{opt.}}$-th HAP transmission power $Tx_{{\mathrm{HAP}}_{j}}=P_{s,3}^{i,j}$ to satellite;12: Wait for *data(k+1)*;13:**end if**14:**end if**


## 6. Simulations and Results

In this section, we present several sets of simulation results to evaluate the effectiveness of the proposed energy-efficient transmissions under a multiple-user, multiple-HAP and single-satellite integrated network. We consider a realistic disaster scenario with a medium-sized city with a 60-km diameter. A natural disaster, such as an earthquake, destroys the terrestrial communication network and the electrical network distribution infrastructure. An IHS network is deployed for public safety personnel and first responders in order to coordinate rescue and first-aid services for the survivors. These simulations were carried out using the NS2 simulator. All of the simulations were run 100 times and the average given.

### 6.1. Evaluation of a Single Terminal

Within the whole network, the devices and terminals work independently without cooperation under frequency division multiple access (FDMA), since the HAPs and the satellite have sufficiently covered the whole disaster area. Cooperation between the devices and terminals will lead to an undesirable complicated electromagnetic environment with additional interference. In this scenario, evaluation of an *i*-th terminal can be used to infer the energy efficiency of transmissions across all terminals, to a certain extent. Since the simulation of one terminal can be performed relatively easily, we evaluate the performance of the energy-efficient transmission strategies at a single terminal as a first step. We assume that four HAPs are deployed and that the *i*-th terminal is randomly located. Table 2 lists the elements of the parameters used in simulations and their values.

**Figure 2:**
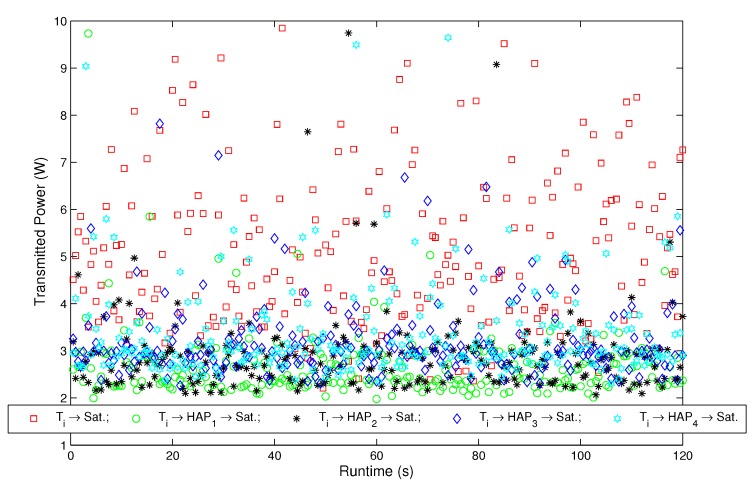
Transmitted power calculated by the direct and relay links *versus* runtime, before applying the energy-efficient path selection.

The Rician factors of channels $h_{1}$ and $h_{2}$ are $\kappa_{1}=12\;\mathrm{dB}$ and $\kappa_{2}=7\;\mathrm{dB}$. The thermal noise power is equal to -207dB. The back-off of the output power for the HAPs and satellite is $[BO_{o}]=3\;\mathrm{dB}$. During the periodic LSA broadcast, each HAP and satellite broadcasts its address (32 bits), geographic position (longitude ψ and latitude ϕ, 64 bits), power $P_{s}$ (32 bits) and antenna gain $G_{s}$ (32 bits) to all users. A total simulation time of 120 s is assumed in this subsection. The *i*-th terminal, *i.e.*, $T_{i}$, sends 772 Kbits of data every Δt=500ms; the packet size of the LSA is 160 bits, and the total time taken for the LSA transmission $T_{LSA}$ is 100 ms. The transmitted power calculated by the direct and relay links *versus* the simulation time before applying the energy-efficient path selection is shown in Figure 2.

**Table 2:** Parameters used in the simulations and their values.

Parameter	Value
Alt.of GEO Sat.	35,786 km
Gain of Sat. $G_{3}$	48.2 dB
Lon.$\psi_{s}$ and Lat. $\phi_{s}$ of GEO Sat.	(143${}^{{}^{\circ}}$31${}^{\prime }$ E, 0${}^{\prime }$ N)
Lon. Vector $\psi_{H}$ of HAP	(118${}^{{}^{\circ}}$08${}^{\prime }$ E, 118${}^{{}^{\circ}}$38${}^{\prime }$ E, 118${}^{{}^{\circ}}$41${}^{\prime }$ E, 118${}^{{}^{\circ}}$23${}^{\prime }$ E)
Lat. Vector $\phi_{H}$ of HAP	(39${}^{{}^{\circ}}$50${}^{\prime }$ N, 39${}^{{}^{\circ}}$51${}^{\prime }$ N, 39${}^{{}^{\circ}}$55${}^{\prime }$ N, 40${}^{{}^{\circ}}$02${}^{\prime }$ N)
Alt. of HAP	22 km
Lon. $\psi_{c}$ and Lat. $\phi_{c}$ of city	(118${}^{{}^{\circ}}$31${}^{\prime }$ E, 39${}^{{}^{\circ}}$56${}^{\prime }$ N)
Alt. of city	200 m
Lon. $\psi_{T}$ and Lat. $\phi_{T}$ of terminal	(118${}^{{}^{\circ}}$22${}^{\prime }$ E, 39${}^{{}^{\circ}}$41${}^{\prime }$ N)
Power required for terminal reception	150 mW
Power required for HAP reception	150 mW
Data rate $R_{b}$	1.544 Mbps
Carrier frequency of HAP↔Sat. link	48 GHz
Carrier frequency of $T_{i}↔HAP$, $T_{i}↔Sat.$ links	3.4 GHz
Gain of HAP↔Sat. antennas $G_{s,2}$	42.3 dB
Gain of terminal antenna $G_{1}$	23.7 dB
Gain of $HAP\to T_{i}$ antennas $G_{r,2}$	29.1 dB
Vector of path loss exponents n	$\left(2.2,2.9,2\right)$

Figure 2 shows the transmission power calculated by direct and relay links *versus* runtime, before applying the energy-efficient path selection. In Figure 2, $T_{i}\to Sat.$ denotes that the terminal only selects the T-S direct link. $T_{i}\to HAP_{1}\to Sat.$, $T_{i}\to HAP_{2}\to Sat.$, $T_{i}\to HAP_{3}\to Sat.$ and $T_{i}\to HAP_{4}\to Sat.$ denote that the terminal only selects the first HAP, the second HAP, the third HAP and the fourth HAP as a relay, respectively. For the T-S direct link, the transmission power is $P_{s,1}\left(t\right)$, and for the T-H-S path, the transmission power is $P_{r}\left(t\right)=P_{s,2}\left(t\right)+P_{s,3}\left(t\right)$. From Figure 2, we can observe that $P_{r}\left(t\right)$ is lower than $P_{s,1}\left(t\right)$ for most of the time slots within the runtime. Therefore, there is an opportunity to reduce the overall transmission power through adaptive path selection. Furthermore, we can also infer that the overall average transmission power of the optimized transmission path will be lower than that of all five paths in Figure 2.

**Figure 3:**
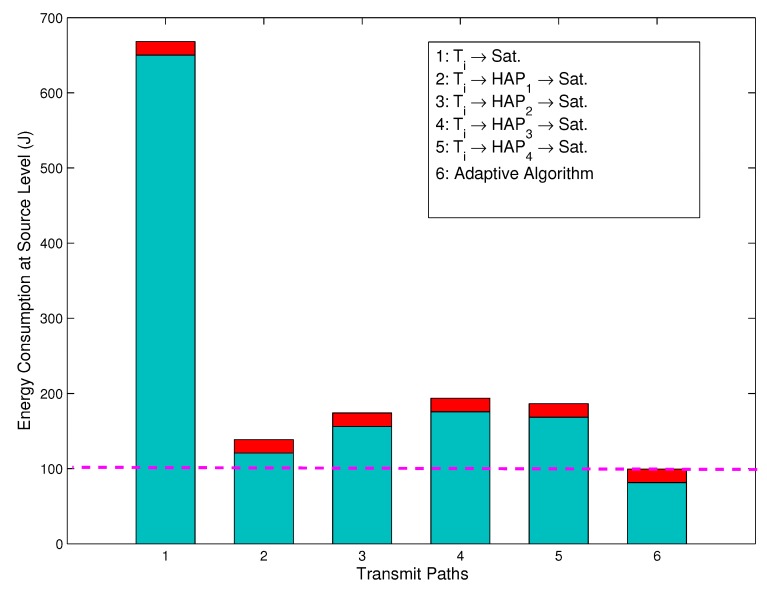
Energy consumption for the direct path, relay paths and adaptive path selection at the source level.

Figure 3 shows the energy consumption for the direct path, relay paths and adaptive path selection at the source level. The energy consumption at the source level is defined as the overall energy consumption at the terminal end. In Figure 3, the term adaptive algorithm denotes that the terminal selects the path based on the proposed adaptive energy-efficient path selection algorithm. We can observe that the energy consumption of the *i*-th terminal for the $T_{i}\to HAP_{1}\to Sat.$ path, $T_{i}\to HAP_{2}\to Sat.$ path, $T_{i}\to HAP_{3}\to Sat.$ path, $T_{i}\to HAP_{4}\to Sat.$ path and adaptive algorithm path is 78.99%,73.62%,69.19%,69.98% and 84.36% lower than that for the $T_{i}\to Sat.$ path. Therefore, the relay path can significantly reduce the terminal energy consumption compared to the direct link, and the terminal energy consumption of the path using the adaptive algorithm is the lowest. From Figure 3, it can be further observed that the terminal energy consumption to process the broadcast LSAs is a little increased (the upper red sub-bars in Figure 3), although the adaptive algorithm is employed. This is due to the proposed LSA concept, which estimates the link state information by broadcasting LSAs in the HAP end and satellite end rather than sensing it at the terminal end. The terminal only receives a tiny quantity of the LSA packets, which can significantly reduce the energy consumption. This is especially applicable for slow flat Rician fading channels.

**Figure 4:**
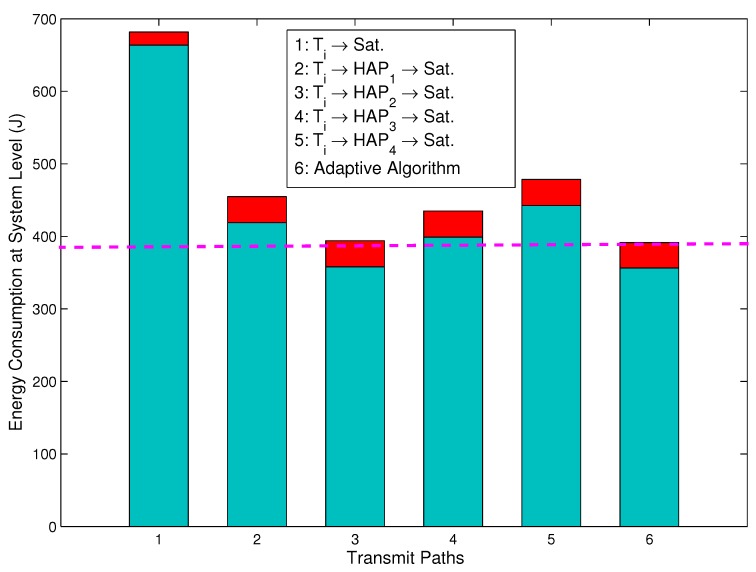
Total energy consumption for the direct link, relay links and adaptive path selection at the system level.

Figure 4 depicts the total energy consumption for the direct link, relay links and adaptive path selection at the system level, where the total energy consumption at the system level is the sum of the energy consumption at both the terminals and the HAPs, and the energy consumption of the satellite is not included. For the T-S direct path, the energy consumption at the system level is equal to that at the source level. However, for the T-H-S relay path, the energy consumption at the system level consists of the energy consumption by the terminals and the energy consumption by the HAPs. From Figure 4, it can be observed that the energy consumption at the system level for the adaptive algorithm path is 43.34%,19.54%,2.25%,13.81%and22.44% lower than that for the $T_{i}\to Sat.$ path, $T_{i}\to HAP_{1}\to Sat.$ path, $T_{i}\to HAP_{2}\to Sat.$ path, $T_{i}\to HAP_{3}\to Sat.$ path and $T_{i}\to HAP_{4}\to Sat.$ path, respectively. Therefore, this implies that the energy consumption of the adaptive algorithm path is not only lower than that of the direct path, but also obviously superior to that of the other relay paths. As shown in Figure 4, we can further observe that the energy consumption of the LSAs for the relay link (the upper red sub-bars in Figure 4) is higher than that for the direct link. Comparing Figure 3 to Figure 4, it can be concluded that the additional energy consumption is mainly due to the HAPs broadcasting LSAs. Although the energy consumption of the LSAs is higher, the reduced energy consumption by the LSAs is much larger than that on their own. Therefore, the path selection method proposed in this paper is energy efficient.

### 6.2. Evaluation of the Multiple Terminals

In this subsection, we mainly investigate the performance of the proposed energy-efficient transmission strategies *versus* the number of terminals and HAPs, *i.e.*, a multiple-user, multiple-HAP and single-satellite integrated network. Then, the energy consumption for the various Rician factors of the T-H links, which are led by the distribution of the multiple terminals, is provided. The same settings for the simulations are used as in the previous subsection, except where stated.

**Figure 5:**
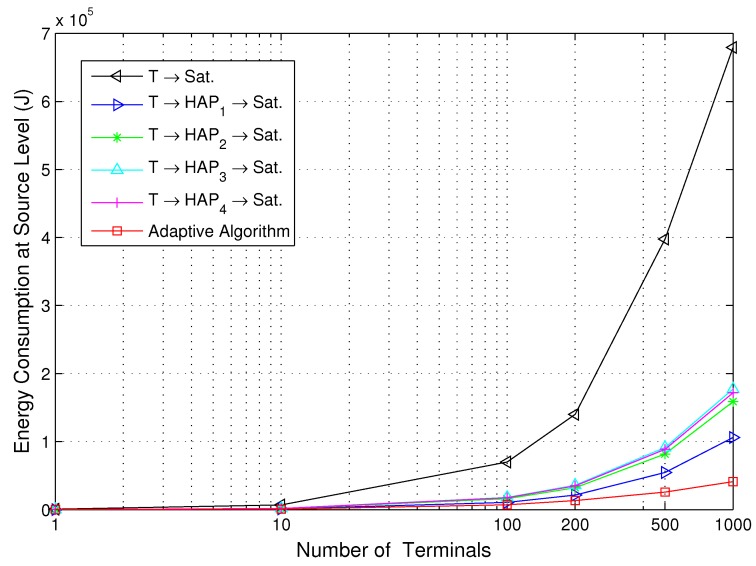
Energy consumption for the direct path, relay paths and adaptive path selection *versus* the number of terminals at the source level.

**Figure 6:**
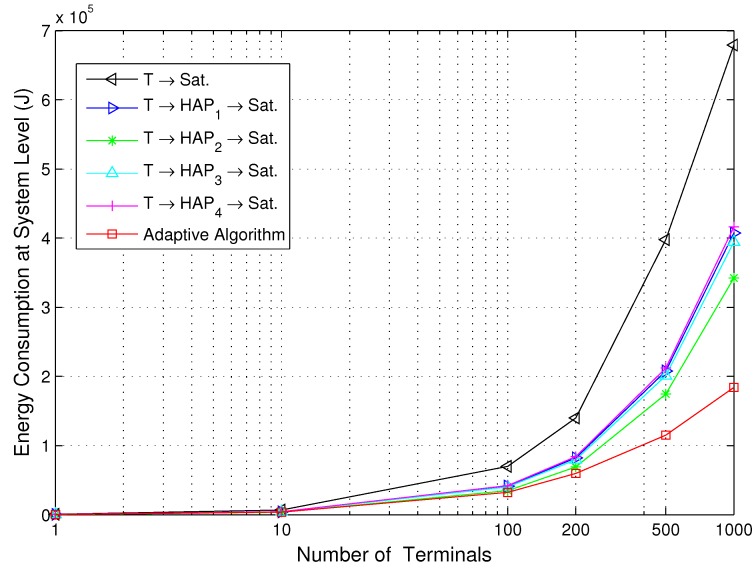
Energy consumption for the direct path, relay paths and adaptive path selection *versus* the number of terminals at the system level.

Figure 5 and Figure 6 show the energy consumption for the direct path, relay paths and adaptive path selection with the various number of terminals at the source and system level, respectively. The locations of the added terminals obey a uniform distribution in the disaster area. The number of terminals used is 1, 10, 100, 200, 500 and 1000. We can observe that the energy consumption of the energy-efficient transmission strategy based on the proposed adaptive algorithm saves energy compared to the other five transmission strategies, and the performance of the T-S link is the worst. This is due to the proposed strategy tending to select the path with the lowest energy consumption during all of the transmission slots. For the T-S link, there is only one channel with Rician fading, serious free-space loss, due to the remote communication distance between the terminal and satellite. The energy consumption is obviously high. We also observe that the performance of all four T-H-S relay links is adjacent, since the four HAPs play a relatively equal role over the disaster area. As the number of terminals is increased, the energy consumption of the proposed adaptive algorithm increases mildly at both the source and system level, which implies that the proposed adaptive algorithm is energy-efficient especially for a multiple-user scenario. Comparing Figure 5 to Figure 6, it can be concluded that the increase in energy consumption at the terminals is less than the overall system energy consumption, which indicates that the proposed adaptive algorithm is efficient for battery-operated remote sensor devices.

**Figure 7:**
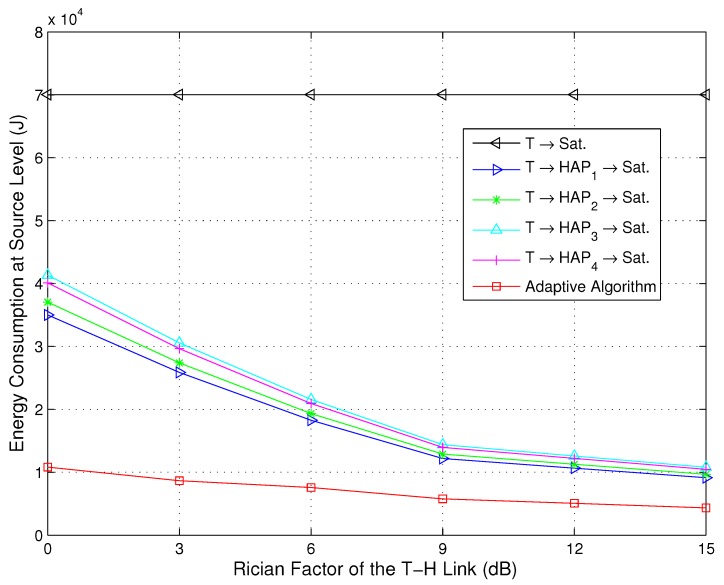
Energy consumption for the direct path, relay paths and adaptive path selection *versus* the Rician factor at the source level ($N_{T}=100,\kappa_{1}=12$ dB).

**Figure 8:**
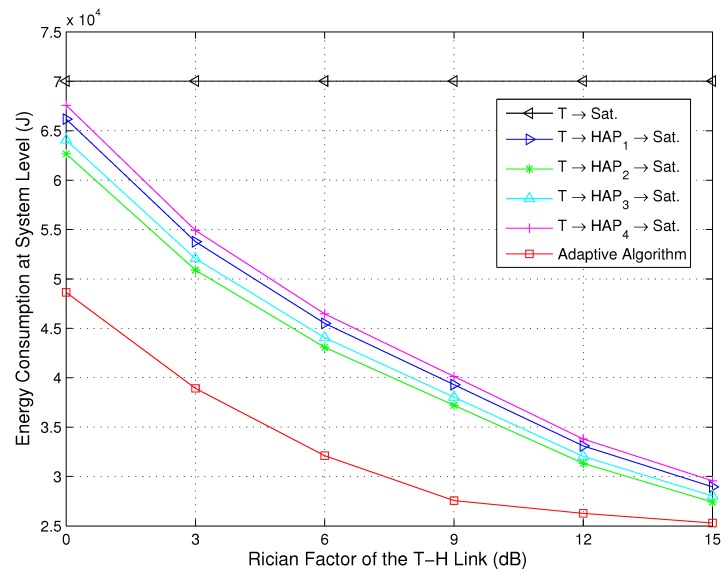
Energy consumption for the direct path, relay paths and adaptive path selection *versus* the Rician factor at the system level ($N_{T}=100,\kappa_{1}=12$ dB).

Since the elevation angles of different terminals for the T-H relay links are various and obstruction by buildings and other tall objects of $h_{2}$ is serious, the Rician factors may change over the disaster area. Figure 7 and Figure 8 depict the energy consumption for the direct path, relay paths and adaptive path selection *versus* the Rician factor of the T-H relay links at the source and system level, respectively. The values of Rician factors for the T-H links are 0 dB, 3 dB, 6 dB, 9 dB, 12 dB and 15 dB, and the value of the Rician factor for the T-S link is maintained at 12 dB. We can observe from Figure 7 that the energy consumption of the proposed adaptive algorithm remains low even with a relatively poor channel, *i.e.*, the value of the Rician factor is small. This is due to the sensor devices receiving and processing the LSAs during the transmission slots. An optimal transmission power $P_{r}^{opt.,i,j}\left(t\right)$ via an optimal transmission path $v_{i}^{{}_{opt.}}$ can be selected based on Algorithm 1. When the channels are relatively poor, the probability of channels with mild fading is small. However, as long as this channel exists, there will be an opportunity to save energy. Therefore, it also indicates that the proposed adaptive algorithm is more energy efficient, particularly in severe channel conditions. We can observe from Figure 8 that the overall energy consumption of the relay transmission strategies is lower than that of the direct strategy, especially for the proposed adaptive algorithm. As the value of the Rician factor is increased, all of the energy consumption of the relay transmission strategies is reduced, but the energy consumption of the proposed adaptive algorithm is still the lowest. This is due to the fact that when the channels are relatively good, the probability of channels with mild fading is large. The transmission without path selection also exhibits a large probability to experience mild fading, so the gaps of the energy consumption among the direct path, relay paths and the adaptive path selection are reduced. However, there is still superiority of the adaptive path selection since the optimal transmission power $P_{r}^{opt.,i,j}\left(t\right)$ and an optimal transmission path $v_{i}^{{}_{opt.}}$ are performed during the whole transmission.

**Figure 9:**
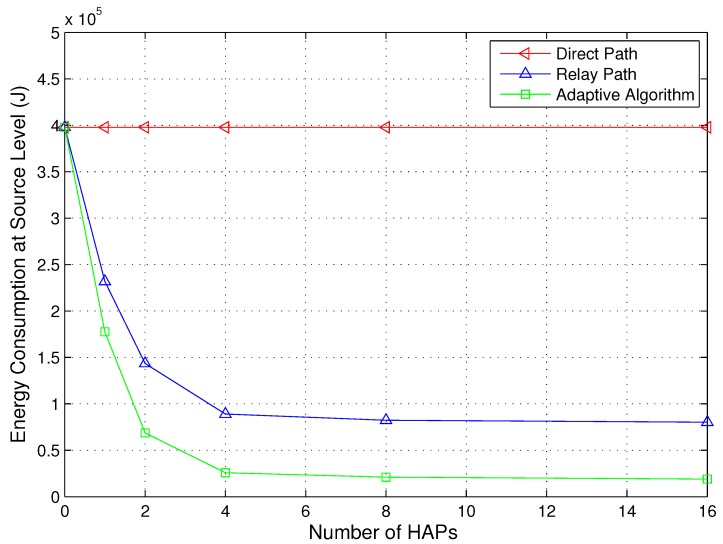
Energy consumption for the direct path, relay paths and adaptive path selection *versus* the number of HAPs at the source level ($N_{T}=500,\kappa_{1}=12$ dB, $\kappa_{2}=7$ dB).

In the simulations above, the number of HAPs is maintained at four. A more practical IHS scenario is that the number of HAPs is nondeterministic. Therefore, we also investigate the energy consumption *versus* the number of HAPs. The number of HAPs used is 0, 1, 2, 4, 8 and 16. We assume that the locations of the added HAPs obey a uniform distribution in the disaster area. Figure 9 depicts the energy consumption *versus* the number of HAPs with $\kappa_{1}=12\;\mathrm{dB}$, $\kappa_{2}=7\;\mathrm{dB}$ and $N_{T}=500$ at the source level. The energy consumption of the relay path is calculated as the average energy consumption of all possible relays. We can observe from Figure 9 that the energy consumption of the relay path and proposed adaptive algorithm decreases as the number of HAPs is increased. The adaptive algorithm is more energy-efficient than the direct path and the relay path. When the value of $N_{H}$ is small (e.g., zero or one), the added HAPs can significantly reduce the energy consumption. However, when the value of $N_{H}$ is large (e.g., eight or 16), the added HAPs can also reduce the energy consumption, but indistinctively. Therefore, permanently increasing the number of HAPs is not necessary. On the contrary, the small number of HAPs will lead to a cost-effective system. Additionally, Table 3 summarizes the saving of energy *versus* the number of HAPs with $\kappa_{1}=12\;\mathrm{dB}$, $\kappa_{2}=7\;\mathrm{dB}$ and $N_{T}=500$ at the system level. The unit of the energy is Joules in Table 3.

**Table 3:** The saving of energy *versus* the number of HAPs at the system level ($N_{T}\;=\;500,\kappa_{1}=12\;\mathrm{dB},\kappa_{2}=7\;\mathrm{dB}$).

	$N_{H}$	0	1	2	4	8	16
Strategies							
Direct Path		0	0	0	0	0	0
Relay Path		0	$1.12\times 10^{5}$	$1.68\times 10^{5}$	$1.98\times 10^{5}$	$2.01\times 10^{5}$	$2.02\times 10^{5}$
Adaptive Path		0	$1.32\times 10^{5}$	$2.27\times 10^{5}$	$2.82\times 10^{5}$	$2.85\times 10^{5}$	$2.87\times 10^{5}$

## 7. Conclusions

This paper has investigated energy-efficient transmission strategies in IHS networks for emergency communications. Firstly, a system model has been established, which consists of a space segment, a near space segment and a ground segment. Secondly, assuming that the channel exhibits a slow flat Rician fading, the concept of LSA has been proposed. The optimal transmission powers ${\hat{P}}_{s,1}$, ${\hat{P}}_{s,2}$ and ${\hat{P}}_{s,3}$ have been derived using the mathematical methods of gradient descent and differential equations. In addition, energy consumption has been modeled, and each step of the adaptive algorithm for the energy-efficient path selection has been described. Finally, the simulations have further proven that the proposed algorithm can improve the quality of communication and reduce the energy consumption. Most significantly, the simulation results show that, at the source level, the energy consumption of the *i*-th terminal for the $T_{i}\to HAP_{1}\to Sat.$ path, $T_{i}\to HAP_{2}\to Sat.$ path, $T_{i}\to HAP_{3}\to Sat.$ path, $T_{i}\;\to \;HAP_{4}\;\to Sat.$ path and the adaptive algorithm path is 78.99%,73.62%,69.19%,69.98%,and84.36% lower, respectively, than that for the $T_{i}\to Sat.$ link. We also found that, at the system level, the energy consumption for the adaptive algorithm path is 43.34%,19.54%,2.25%,13.81%and22.44% lower than that for the $T_{i}\to Sat.$ path, $T_{i}\;\to \;HAP_{1}\;\to \;Sat.$ path, $T_{i}\;\to \;HAP_{2}\to Sat.$ path, $T_{i}\;\to \;HAP_{3}\to Sat.$ path and the $T_{i}\;\to \;HAP_{4}\;\to Sat.$ path, respectively. Additionally, in a multiple-user, multiple-HAP and single-satellite scenario, the simulation results also show that the proposed adaptive algorithm is energy efficient with various numbers of terminals, numbers of HAPs and Rician factors. We conclude that the proposed energy-efficient transmissions and adaptive path selection algorithm are promising solutions for remote WSNs for emergency scenarios. In the paper, a deterministic method is used to formulate the solution. However, a more reliable and interesting method is to estimate the parameters in the system using Bayesian or Kalman filtering (KF) at the terminal. Therefore, further studies will extend the results in a nondeterministic way.

## References

[1] Celandroni N., Ferro E., Gotta A., Oligeri G., Roseti C., Luglio M., Bisio I., Cello M., Davoli F., Panagopoulos A.D. (2013). A survey of architectures and scenarios in satellite-based wireless sensor networks: System design aspects. Int. J. Sat. Commun. Netw..

[2] Baldini G., Karanasios S., Allen D., Vergari F. (2014). Survey of wireless communication technologies for public safety. IEEE Commun. Surv. Tutor..

[3] Dong F., Li M., Gong X., Li H., Gao F. (2015). Diversity performance analysis on multiple HAP networks. Sensors.

[4] Wang W., Jiang D. (2014). Integrated wireless sensor systems via near-space and satellite platforms: A review. IEEE Sens. J..

[5] Chen L., Jia X., Meng L., Wang L. (2013). Expedite privacy-preserving emergency communication scheme for VANETs. Int. J. Distrib. Sens. Netw..

[6] Dong F., Huang Q., Li H., Kong B., Zhang W. (2014). A novel M2M backbone network architecture. Int. J. Distrib. Sens. Netw..

[7] Mohammed A., Mehmood A., Pavlidou F.-N., Mohorcic M. (2011). The role of high-altitude platforms (HAPs) in the global wireless connectivity. IEEE Proc..

[8] Dong F., Lv J., Gong X., Li C. (2014). Optimization design of structure invulnerability in space information network. J. Commun..

[9] Grace D., Mohorcic M. (2011). Broadband Communications via High-Altitude Platforms.

[10] Fan Z., Sun H., Wang L. (2015). Research of the classification model based on dominance rough set approach for China emergency communication. Math. Probl. Eng..

[11] Karapantazis S., Pavlidou F. (2005). Broadband communications via high-altitude platforms: A survey. IEEE Commun. Surv. Tutor..

[12] Casoni M., Grazia C., Klapez M., Patriciello N., Amditis A., Sdongos E. (2015). Integration of satellite and LTE for disaster recovery. IEEE Commun. Mag..

[13] Deaton J.D. (2008). High altitude platforms for disaster recovery: Capabilities, strategies, and techniques for emergency telecommunications. EURASIP J. Wirel. Commun. Netw..

[14] Asensio Á., Blanco T., Blasco R., Marco Á., Casas R. (2015). Managing emergency situations in the smart city: The smart signal. Sensors.

[15] Aranti S., Sanctis M.D., Spinella S.C., Monti M., Cianca E., Molinaro A., Iera A., Ruggieri M. Hybrid System HAP-WiFi for Incident Area Networks. Proceedings of the ICST Personal Satellite Services.

[16] Lin C.Y., Chu E.T.-H., Ku L.W., Liu J.W. (2014). Active disaster response system for a amart building. Sensors.

[17] Elhawary M., Haas Z.J. (2011). Energy-efficient protocol for cooperative networks. IEEE ACM Trans. Netw..

[18] Nasim M., Qaisar S., Lee S. (2012). An energy efficient cooperative hierarchical MIMO clustering scheme for wireless sensor networks. Sensors.

[19] Kandeepan S., Gomez K., Reynaud L., Rasheed T. (2014). Aerial-terrestrial communications: Terrestrial cooperation and energy-efficient transmissions to aerial base stations. IEEE Trans. Aerosp. Electron. Syst..

[20] Alagoz F., Gür G. (2011). Energy efficiency and satellite networking: A holistic overview. IEEE Proc..

[21] Zhu Y., Xin Y., Kam P.-Y. (2008). Optimal transmission strategies for rayleigh fading relay channels. IEEE Trans. Wirel. Commun..

[22] Madan R., Mehta N.B., Molisch A.F., Zhang J. (2008). Energy-efficient cooperative relaying over fading channels with simple relay selection. IEEE Trans. Wirel. Commun..

[23] Dovis F., Fantini R., Mondin M., Savi P. (2002). Small-scale fading for high-altitude platform (HAP) propagation channels. IEEE J. Sel. Areas Commun..

[24] Supnithi P., Wongtrairat W., Tantaratana S. (2009). Performance of M-PSK in mobile satellite communication over combined ionospheric scintillation and flat fading channels with MRC diversity. IEEE Trans. Wirel. Commun..

[25] Zhu L., Yan X., Zhu Y. High Altitude Platform-based Two-hop Relaying Emergency Communications Schemes. Proceedings of the 5th International Conference on Wireless Communication, Network and Mobile Computing (WiCom’09).

[26] Wang X., Gao X., Zong R. Energy-Efficient Deployment of Airships for High Altitude Platforms: A Deterministic Annealing Approach. Proceedings of the IEEE Global Telecommunications Conference (GLOBECOM’11).

[27] Luglio M., Theodoridis G., Roseti C., Pavlidou N. (2009). A TCP driven CAC scheme: Efficient resource utilization in a leaky HAP-satellite integrated scenario. IEEE Trans. Aerosp. Electron. Syst..

[28] Pace P., Aloi G. (2008). Disaster monitoring and mitigation using aerospace technologies and integrated telecommunication networks. IEEE Aerosp. Electron. Syst. Mag..

[29] Wang H., Liu A., Pan X., Yang J. (2014). Optimization of power allocation for multiusers in multi-spot-beam satellite communication systems. Math. Probl. Eng..

[30] Maleki S., Chatzinotas S., Evans B., Liolis K., Grotz J., Vanelli-Coralli A., Chuberre N. (2015). Cognitive spectrum utilization in Ka band multibeam satellite communications. IEEE Commun. Mag..

[31] Dong F., He Y., Zhou X., Yao Q. Optimization and Design of HAP Broadband Communication Networks. Proceedings of the IEEE 5th International Conference on Information Science and Technology (ICIST’15).

[32] Aziz M.R.K., Iskandar A.A. Channel Estimation for LTE Downlink in High Altitude Platforms (HAPs) Systems. Proceedings of the International Conference on Information and Communication Technology (ICoICT’13).

[33] Andreev S., Gerasimenko M., Galinina O., Koucheryavy Y., Himayat N., Yeh S.-P., Talwar S. (2014). Intelligent access network selection in converged multi-radio heterogeneous networks. IEEE Wirel. Commun..

[34] Lutz E., Cygan D., Dippold M., Dolainsky F., Papke W. (1991). The land mobile satellite communication channel-recording, statistics, and channel model. IEEE Trans. Veh. Technol..

[35] Rappaport T. S. (1996). Wireless Communications: Principles and Practice.

[36] Hatsuda T., Hashimoto K., Masuda J., Murakami J. (2006). Diversity systems comparison of satellite visibility improvement for designing mobile broadcasting satellite system. IEEE Trans. Antennas Propag..

